# Circulating microparticles in severe pulmonary arterial hypertension increase intercellular adhesion molecule-1 expression selectively in pulmonary artery endothelium

**DOI:** 10.1186/s12931-016-0445-1

**Published:** 2016-10-20

**Authors:** Leslie A. Blair, April K. Haven, Natalie N. Bauer

**Affiliations:** 1 Department of Pharmacology, College of Medicine, University of South Alabama, 5851 USA Drive North, MSB 3340, Mobile, AL 36688 USA; 2 Center for Lung Biology, College of Medicine, University of South Alabama, 5851 USA Drive North, MSB 3340, Mobile, AL 36688 USA

**Keywords:** Microparticle, Pulmonary hypertension, Inflammation, Endothelium

## Abstract

**Background:**

Microparticles (MPs) stimulate inflammatory adhesion molecule expression in systemic vascular diseases, however it is unknown whether circulating MPs stimulate localized ICAM-1 expression in the heterogeneically distinct pulmonary endothelium during pulmonary arterial hypertension (PAH). Pulmonary vascular lesions with infiltrating inflammatory cells in PAH form in the pulmonary arteries and arterioles, but not the microcirculation. Therefore, we sought to determine whether circulating MPs from PAH stimulate pulmonary artery endothelial cell-selective ICAM-1 expression.

**Results:**

Pulmonary artery endothelial cells (PAECs) were exposed to MPs isolated from the circulation of a rat model of severe PAH. During late-stage (8-weeks) PAH, but not early-stage (3-weeks), an increase in ICAM-1 was observed. To determine whether PAH MP-induced ICAM-1 was selective for a specific segment of the pulmonary circulation, pulmonary microvascular endothelial cells (PMVECs) were exposed to late-stage PAH MPs and no increase in ICAM-1 was detected. A select population of circulating MPs, the late-stage endoglin + MPs, were used to assess their ability to stimulate ICAM-1 and it was determined that the endoglin + MPs were sufficient to promote ICAM-1 increases in the whole cell, but not surface only expression.

**Conclusions:**

Late-stage, but not early-stage, MPs in a model of severe PAH selectively induce ICAM-1 in pulmonary artery endothelium, but not pulmonary microcirculation. Further, the selected endoglin + PAH MPs, but not endoglin + MPs from control, are sufficient to promote whole cell ICAM-1 in PAECs. The implications of this work are that MPs in late-stage PAH are capable of inducing ICAM-1 expression selectively in the pulmonary artery. ICAM-1 likely plays a significant role in the observed inflammatory cell recruitment, specifically to vascular lesions in the pulmonary artery and not the pulmonary microcirculation.

**Electronic supplementary material:**

The online version of this article (doi:10.1186/s12931-016-0445-1) contains supplementary material, which is available to authorized users.

## Background

Increased numbers of microparticles (MPs) are evident in pulmonary arterial hypertension (PAH) patients and animal models, and specific populations of the extracellular vesicles, such as endoglin-positive MPs, correlate with disease severity [1–3]. MPs are implicated in pulmonary vasoreactivity and stimulation of vascular remodeling in pulmonary hypertension [4–6]. Recent emphasis has been placed on the importance of infiltrating inflammatory cells in the formation of pulmonary vascular lesions. These cells include macrophages, T and B lymphocytes, and mast cells. However, the mechanisms responsible for the localized transmigration of these inflammatory cells into the perivascular tissue remains unknown [7]. Cellular adhesion molecule expression is enhanced by proinflammatory MPs [8–10]. Further, endothelial inflammation promoted by MPs can lead to enhanced endothelial-leukocyte interactions [8, 10–12]. Thus, we propose that circulating MPs in PAH contribute to the recruitment of leukocytes to specific sites of pulmonary vascular lesion formation.

In the pulmonary circulation, heterogeneity exists between endothelial cells in the macrovascular and microvascular segments. Although they reside in close proximity to one another, pulmonary artery endothelial cells (PAECs) and pulmonary microvascular endothelial cells (PMVECs) are molecularly and functionally distinct [13, 14]. PMVECs create an endothelial monolayer with a more restrictive barrier, and certain agonists are capable of activating PAECs without a measurable effect on PMVECs [14]. It has been assumed that MPs will affect all endothelium in a similar manner, however, based on evidence of endothelial heterogeneity, it is likely the responses to MPs will be vascular site specific.

Cellular adhesion molecules are important for immune cell attachment and migration into lung tissue. The expression of intercellular adhesion molecule-1 (ICAM-1) influences the adhesion of circulating immune cells to the pulmonary endothelium and, therefore, contributes to immune cell migration and perivascular infiltration. ICAM-1 on endothelial cells binds to leukocyte function-associated antigen-1 (LFA-1) and the macrophage-1 antigen (Mac-1). The protein is expressed constitutively on several cell types, but ICAM-1 is increased significantly in response to many proinflammatory signals [15]. Circulating, or soluble, ICAM-1 is increased in patients with PAH as is expression of ICAM-1 on endothelium of pulmonary arteries [16, 17]. Soluble ICAM-1 is present in circulations of healthy individuals, but the levels are increased in pathologies involving endothelial activation. Therapeutics for PAH patients, such as bosentan, reduce elevated levels of soluble ICAM-1 along with other inflammatory cytokines and improve 6-min walk distances [18]. Thus, ICAM-1 and the inflammatory cells recruited to the sites of its expression likely contribute to PAH complications in patients.

Considering increased counts of MPs, the proinflammatory state in PAH, and the heterogeneity of the pulmonary vascular endothelium, the purpose of this study was to determine whether MPs from severe PAH are a proinflammatory stimulus for pulmonary endothelial cells in a segment specific manner. Since the observed pulmonary vascular lesions in PAH are localized to arteries and small arterioles, but not the microcirculation, we examined the response to MPs in the pulmonary artery endothelial cells and pulmonary microvascular endothelial cells. Pulmonary endothelial cells were exposed to MPs from the Sugen/Hypoxia/Normoxia rat model of PAH. We investigated the presence of intercellular adhesion molecule-1 (ICAM-1) in both PAECs and PMVECs to determine if the two cell types had unique responses to circulating PAH MPs.

## Methods

### Animal model

All experimental procedures were approved by the Institutional Animal Care and Use Committee at the University of South Alabama (Protocol # 829408, PI Natalie Bauer; Association for Assessment and Accreditation of Laboratory Animal Care International approved since 1999; Compliance with Public Health Service Policy on Humane Care and Use of Laboratory Animals Assurance Number: A3288-01). Adult male Sprague-Dawley rats (Harlan Laboratories, Inc.) weighing 200–225 g were randomly assigned to one of two treatment groups. Control rats were housed in normoxia (21 % O_2_) for the duration of the experiment. Sugen/Hypoxia/Normoxia rats, which will be referred to as PAH rats, were administered subcutaneous injections of Sugen5416 (20 mg/kg, Cayman) then placed in hypoxia (10 % O_2_) for 3 weeks. The rats were removed from hypoxia and placed in normoxia at the end of the 3 week period. Rats in each group (3–5 per group) were euthanized at one of two experimental time points – 3 or 8 weeks. Heparinized blood was collected from the right ventricle.

### MP isolation

MPs were isolated by a series of centrifugations. Heparinized blood obtained from each rat by cardiac puncture was first separated by centrifugation at 1500 × g for 7 min. The top plasma portion was retained and centrifuged further at 13,000 × g for 10 min to eliminate platelets from the samples. Finally, platelet-free plasma was ultracentrifuged at 100,000 × g, 4 °C for 45 min to pellet submicron vesicles. A small portion of intact MPs from each rat was used to determine the protein concentration prior to in vitro treatments. Briefly, a microLowry protein assay was performed on MPs separated from plasma by ultracentrifugation. Data obtained by this method were used to determine the amount of MP-associated protein per milliliter of plasma from each rat. For treatments, MPs pelleted by ultracentrifugation were resuspended in treatment media (DMEM with 10 % fetal bovine serum devoid of MPs and 1 % penicillin-streptomycin) at a concentration of 50 μg/mL.

### MP sorting

MPs were isolated from blood as previously described. The MP-containing pellet was resuspended in growth media without phenol or serum MPs. To distinguish the population of MPs that contain endoglin on their surfaces, 2 μg of endoglin antibody (Santa Cruz, #sc-19793) conjugated with the APEX Alexa Fluor 488 Antibody labeling kit (Invitrogen, #A10468) was incubated with MPs for 30 min at 37 °C. Finally, samples were sorted into unstained and Endoglin-positive populations using the BD FACSAria III in our institution’s flow cytometry core. In addition to sorting microparticles, the BD FACSAria III counted the submicron particles as they passed through. Total and Endoglin + MP counts per 200 μL of platelet-free plasma were determined from these data.

### Endothelial cell culture and treatments

PAECs and PMVECs from the University of South Alabama’s Center for Lung Biology Cell Culture Core were used for these studies. Cells were maintained at 37 °C in room air plus 5 % CO_2_ with Dulbecco’s Modified Eagle Medium supplemented with 10 % fetal bovine serum and 1 % penicillin-streptomycin. To study the effects of MPs from control and PAH rats on endothelial cells, monolayers of PAECs and PMVECs were washed with PBS then incubated with 50 micrograms of MPs per 1 mL of treatment media for 6 h at 37 °C. Additional monolayers of PAECs and PMVECs were treated in the same manner with treatment media only and with media containing 3 % supernatant from activated pulmonary macrophages (AM supernatant; a generous gift from Dr. Diego Alvarez). Briefly, to generate AM supernatant rat alveolar macrophages (ATCC) are cultured overnight and treated with 100 ng/mL LPS for 18 h. Medium is collected, centrifuged at 500 × g for 5 min and supernatant retained. The supernatant is centrifuged again at 4000 × g for 10 min and filtered through a 0.2 um syringe filter into a sterile tube. Aliquots are stored at −20 °C until use.

### Flow cytometry analysis of endothelial ICAM-1

Following a 6-h incubation period, treatment media with or without MPs was removed from monolayers of PAECs and PMVECs. Cells were washed with PBS, then trypsinized to remove them from the cell culture plate. Centrifugation at 500 × g for 5 min at 4 °C was performed to pellet the cells. For treated cells used for whole cell staining, 0.1 % formaldehyde and methanol fixation buffer (Invitrogen, V25118) was added for 20 min at room temperature to permeabilize the membranes. Intact cells for membrane staining were resuspended in PBS and kept on ice during this time period. Then all cells were pelleted again, washed with PBS, and incubated with a fluorescein-conjugated ICAM-1 antibody for 30 min at 4 °C (R&D systems, FAB5831F). After antibody incubation, cells were centrifuged again at 500 × g for 5 min at 4 °C, washed, then finally resuspended in 2 % formalin in PBS. Fixed PAECs and PMVECs were stored overnight at 4 °C. The following morning samples were run on a BD FACSCanto II flow cytometer to determine the percentage of treated cells that were ICAM-1 positive.

### Statistics

Statistical analyses were performed in GraphPad Prism version 5. All data are presented as means with bars representing the standard error of the means. Two-tailed t-tests were performed when comparing the means of two treatment groups. One-way analysis of variance (ANOVA) tests combined with Tukey’s multiple comparison post-tests were used to determine significance in data sets containing more than two treatment groups. *P*-values less than 0.05 were considered significant.

### Supplemental data methods

#### ICAM-1 immunohistochemistry

Formalin-fixed, parrafin-embedded tissue sections were deparaffinized at 60 °C for 20 min and followed by 2 xylene washes. Sections were rehydrated in a graded series of ethanol followed by distilled water. After rehydration, tissue sections were immersed in preheated citrate buffer (Vector Labs; HC-3300) at 90 °C for 20 min for antigen unmasking. Sections were then washed three times with TBS-T (0.5 % Triton-X) and incubated with Background Buster™ (Innovex; NB306) for 60 min at room temperature to block nonspecific binding. Tissue sections were incubated overnight at 4 °C with ICAM-1 antibody (R & D Systems; FAB58315F) at a dilution of 1:500 in BSA. After additional washing steps, slides were mounted with Ultra Cruz Mounting Medium (sc-24941). Slides were viewed using a Nikon A1R spectral confocal microscope housed in the BioImaging Core Facility.

## Results

### MP counts are elevated in PAH rats

**Fig. 1:**
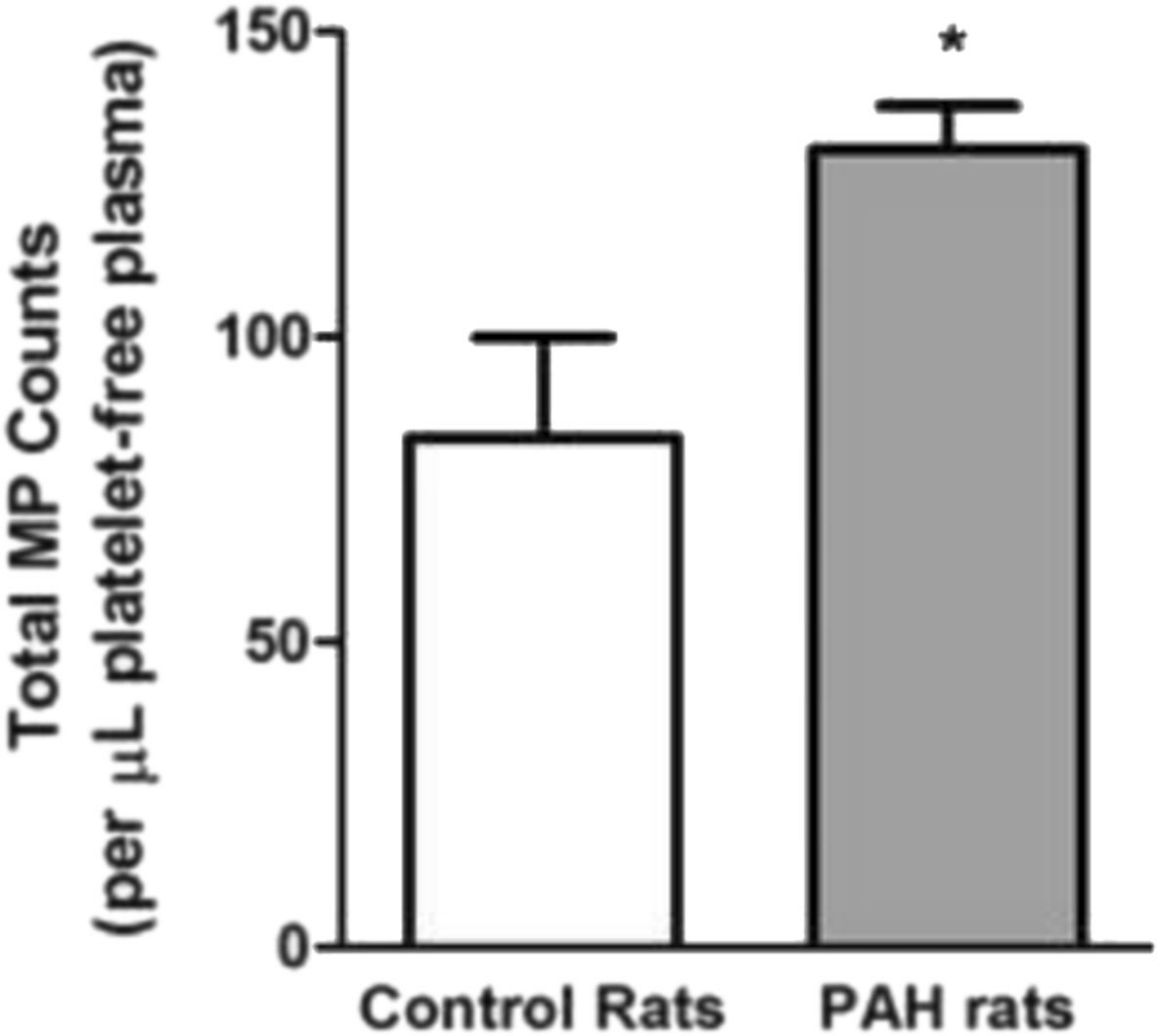
Circulating MP counts are elevated in PAH rats. The number of MPs per microliter of platelet-free plasma was determined using flow cytometry. Compared to 8-week control rats, the number of circulating MPs is significantly greater in 8-week PAH rats. *n* = 5 * = *p* < 0.05

Circulating microparticles are increased in patients with PAH [2, 3]. Therefore, we investigated differences in MP counts between control and 8-week PAH rats. Submicron vesicles were isolated from the same volume of platelet-free plasma from control and PAH rats. Flow cytometry was then used to enumerate total MPs from each sample. We found that PAH rats contain an increased number of circulating MPs per microliter of platelet-free plasma than do control rats (Fig. 1). Thus, our PAH rat model mimics human PAH in the elevated number of circulating MPs.

### MPs from late-stage PAH rats increase ICAM-1 in PAECs

**Fig. 2:**
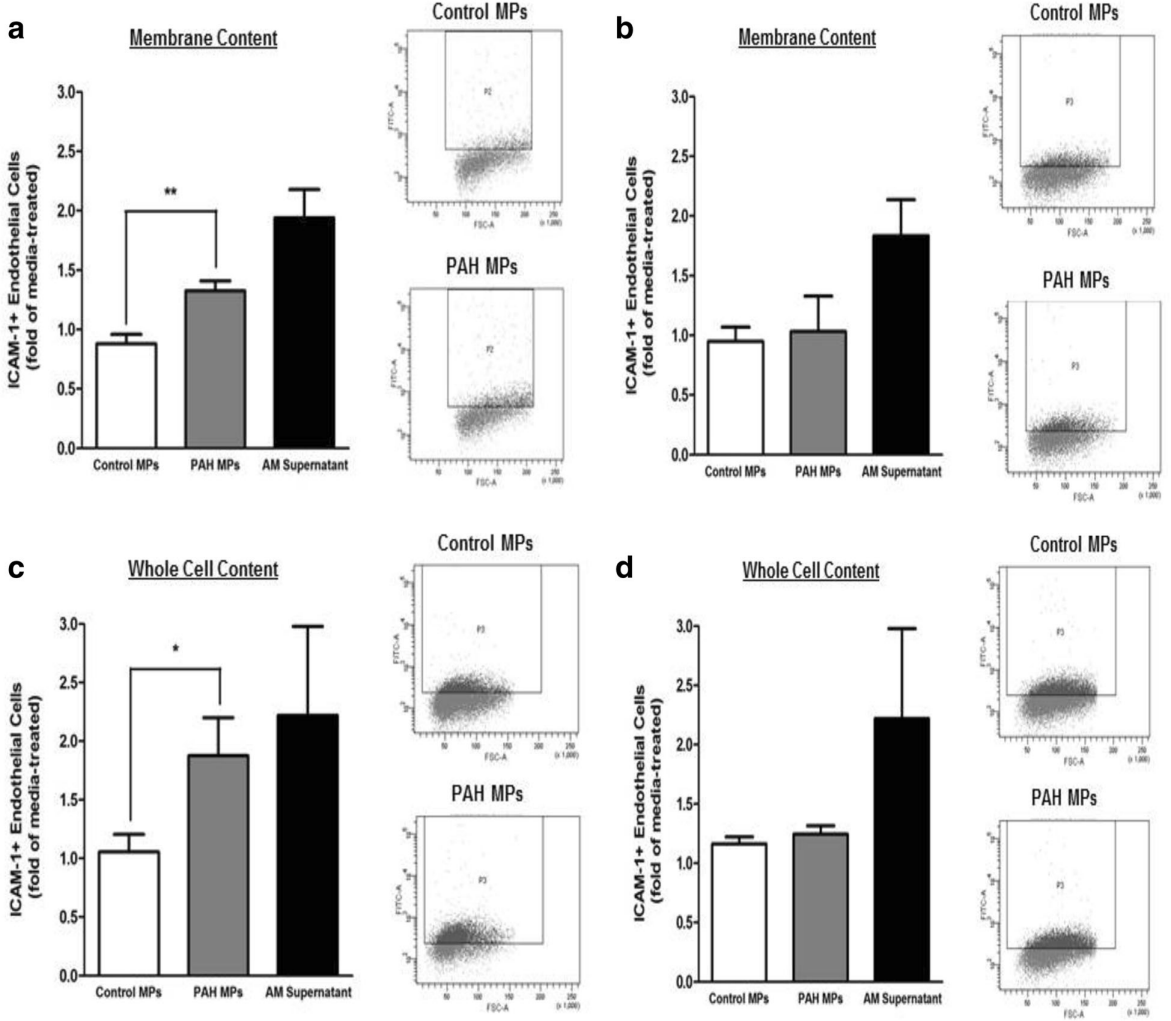
MPs from late-stage PAH rats increase the content of ICAM-1 in PAECs. PAECs were treated with 50ug/mL MPs from age matched control rats, 8-week (**a**), or 3-week (**b**) severe PAH rats. Surface (non-permeabilized cells) ICAM-1 was analyzed by flow cytometry and represented by representative dot plots and fold analysis. Whole cell (permeabilized) content of ICAM-1 was also analyzed (**c** and **d**; 8-week and 3-week, respectively). Eight-week, or late-stage, PAH MPs significantly increased both surface and whole cell ICAM-1 in PAECs, whereas 3-week PAH MPs did not. *n* = 5 * = *p* < 0.05, ** = *p* < 0.01

PAH is a progressively worsening disease and with the advent of a progressive model of lesion formation we have the ability to determine whether early- and late-stage microparticles impact the pulmonary endothelium. Circulating MPs can increase the expression of cellular adhesion molecules in endothelial cells [8, 9]. Thus, we investigated the ability of circulating MPs from a progressive rat model of PAH to increase the expression of ICAM-1 in PAECs. The rat model of PAH used in these studies exhibits hemodynamic and histological characteristics that worsen with time [19]. Additionally, inflammatory cells that accumulate around remodeled pulmonary vessels are dynamic in this animal model [20]. Prior studies have shown that at 8-weeks the hemodynamic profile of this model is severe PAH, the pulmonary vascular lesions range from concentric laminar smooth muscle to fully occluded and we confirmed ICAM-1 expression by staining control and late-stage PAH lung tissues (Additional file 1: Figure S1). By flow cytometry methods, we were able to assess the whole cell (permeabilized cells) and membrane content of ICAM-1 in PAECs treated with MPs from PAH and control rats. For each study we used supernatant from activated alveolar macrophages, containing multiple cytokines, as a positive control for cell responsiveness. We found that PAECs treated with MPs from 8-week, but not the 3-week, PAH rats increase ICAM-1 expression (Fig. 2). MPs from 8-week, but not 3-week, PAH rats enhanced the membrane surface expression of ICAM-1 in PAECs (Figs. 2a and b). PAECs treated with MPs from 8-week PAH rats also exhibited a two-fold increase in ICAM-1 in permeabilized cells (whole cell ICAM-1) compared to those treated with MPs from 8-week control rats, and further, no whole cell change in ICAM-1 was detected with 3-week MPs (Fig. 2c and d). These data suggest that MPs in our late-stage animal model of severe PAH have a significant impact on the inflammatory profile of the pulmonary artery endothelium.

### PMVEC ICAM-1 is not increased by MPs from PAH rats

**Fig. 3:**
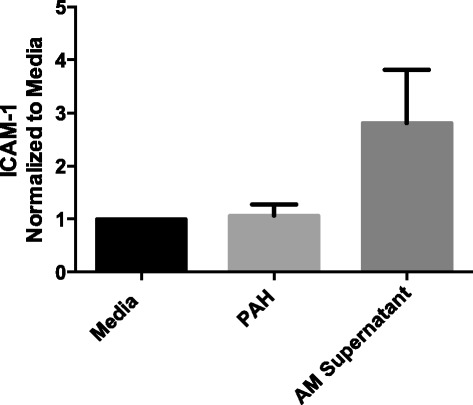
MPs from 8-week PAH rats do not alter ICAM-1 on PMVECs. PMVECs were treated with 50ug/mL MPs from 8-week PAH rats. PMVECs were analyzed by flow cytometry for membrane ICAM-1 and no changes were evident with PAH-MP treatment. As a positive control, AM supernatant did increase ICAM-1 on PMVECs. *n* = 3 * = *p* < 0.05

Within the pulmonary circulation exists significant phenotypic heterogeneity. Pulmonary artery endothelium and pulmonary microvascular endothelium are unique with respect to location and responses to a number of physiologic stimuli [13, 14]. Thus, we examined whether the circulating MPs at 8-weeks stimulated ICAM-1 expression on the PMVECs. We found that there was no increase in ICAM-1 staining of PMVECs treated with PAH MPs (Fig. 3).

### Endoglin-positive MP counts in 8 week PAH rats

**Fig. 4:**
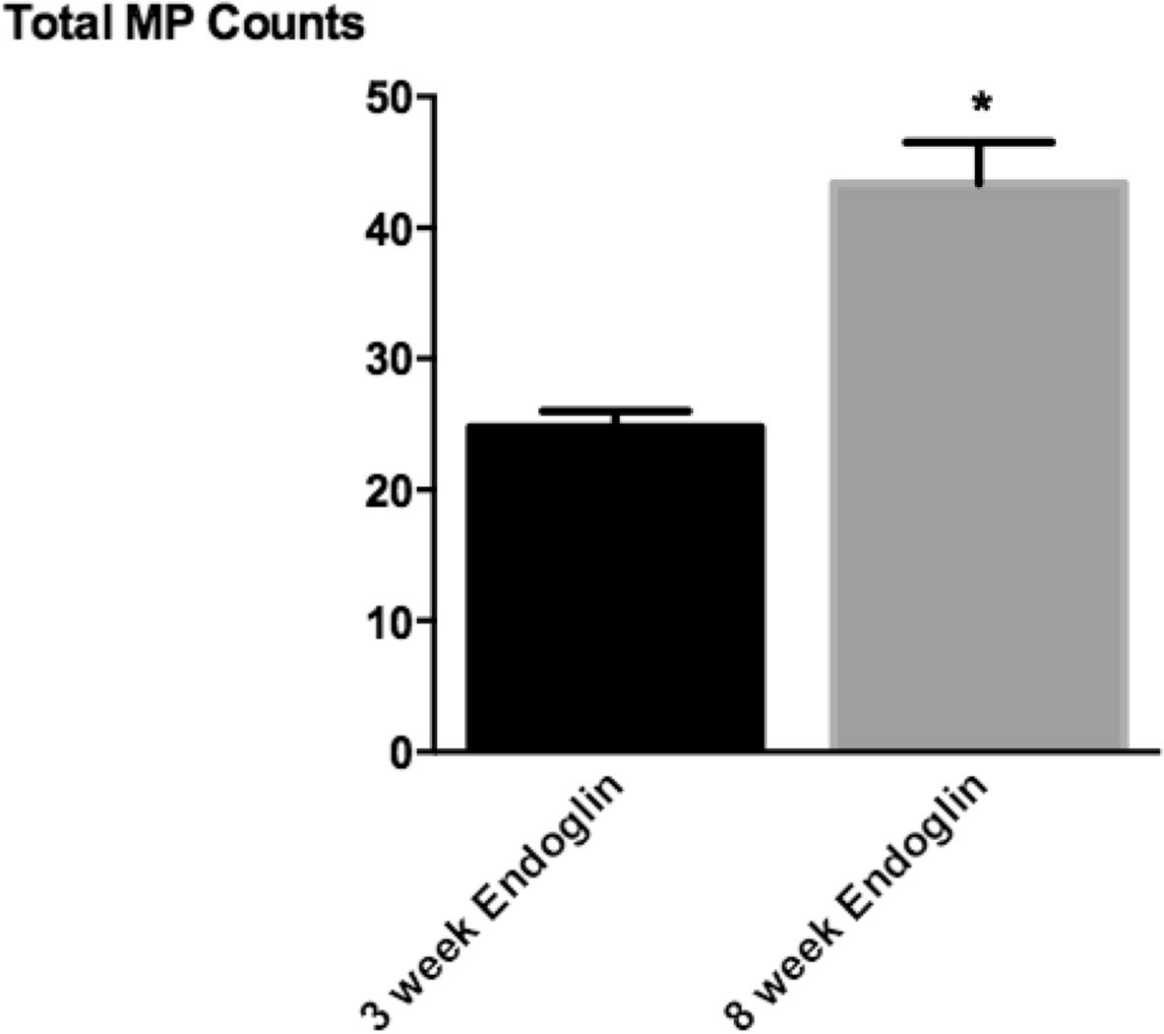
Endoglin + MP counts in severe PAH rats. MPs from 3 to 8-week PAH rats were sorted based on their surface expression of Endoglin, and Endoglin + MPs were counted. We found that there is a significant increase in the number of Endoglin + MPs between 3 and 8-week PAH rats. *n* = 6 * = *p* < 0.05

Endoglin is an accessory receptor for members of the TGF-β receptor superfamily. Endoglin plays a role in PAH associated with haemorrhagic telangiectasia [3]. In examinations of MPs from the circulation of PAH patients, the endoglin-positive population of MPs is increased [3]. We examined whether the percentage of the endoglin-positive MPs increased as the PAH progressed in our rat model. Compared to 3-week animals, the 8-week endoglin-positive MP population increased from 20 % to nearly 50 % of the total population of circulating MPs (Fig. 4).

### Endoglin-positive MPs from late-stage PAH rats drive the increase in whole cell ICAM-1 content in PAECs

**Fig. 5:**
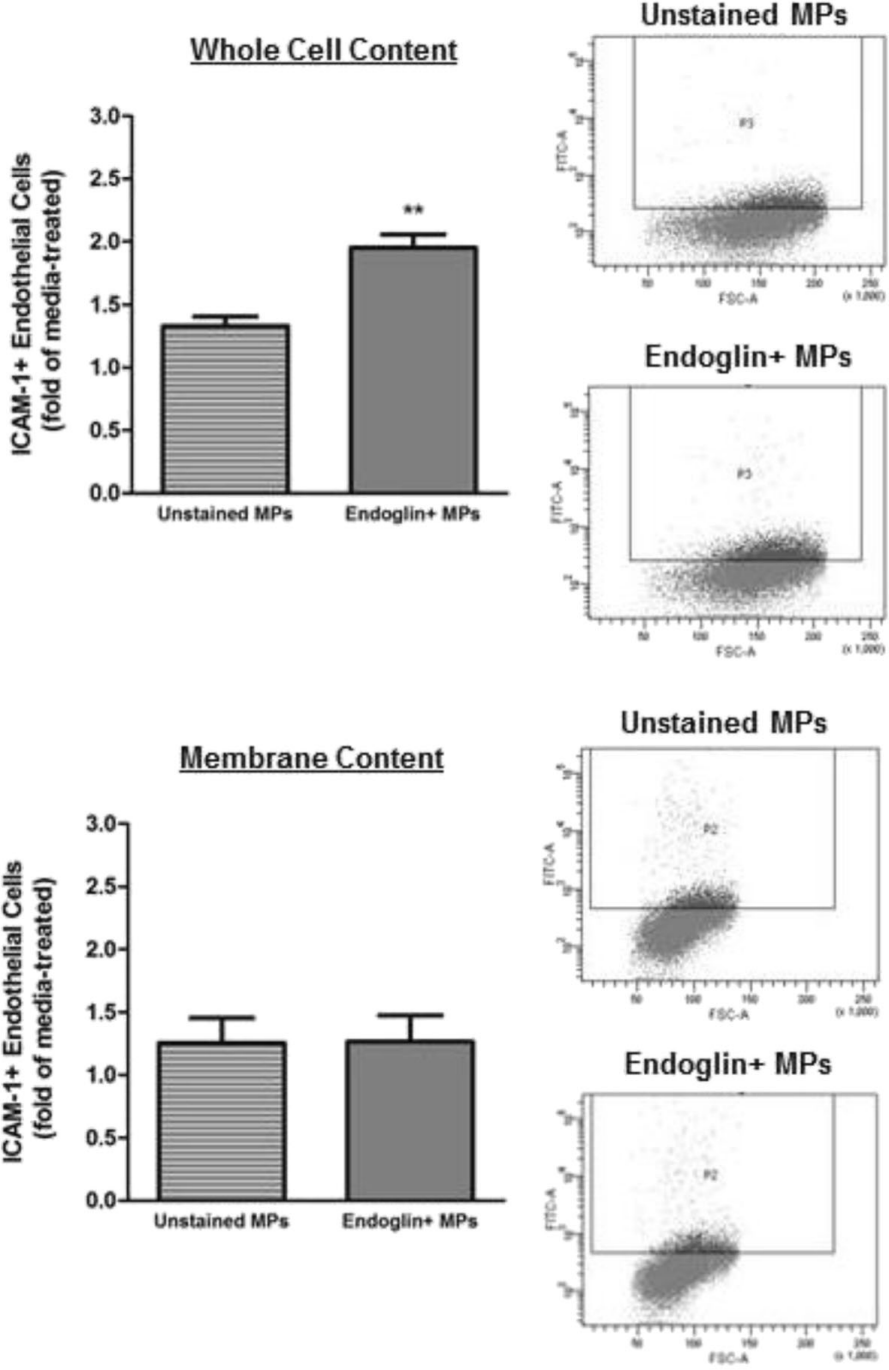
Endoglin + MPs from 8-week PAH rats increase the whole cell content of ICAM-1 in PAECs. Endoglin + MPs stimulate a two-fold increase above media-treated PAECs in whole cell ICAM-1 (**a**, graph in *left panel*), but no significant change in membrane content (**b**). *n* = 4 ** = *p* < 0.01

To determine whether the endoglin + MPs had a direct effect on the PAECs cells we sorted MPs from 8-week PAH animals into endoglin + and endoglin- populations and used each population to treat PAECs to assess the specific effects on ICAM-1 content. Whole cell ICAM-1 was increased in PAECs treated with endoglin + MPs from 8-week PAH rats compared to that in PAECs treated with unstained, or endoglin-, MPs from the same rats (Fig. 5a). Whole cell ICAM-1 levels increased to about two-fold of that of media-treated cells. These data mirror the whole cell increase observed in PAECs treated with the entire population of MPs from 8-week PAH rats. In contrast, membrane ICAM-1 content was not significantly elevated in PAECs treated with endoglin + MPs from 8-week PAH rats (Fig. 5b). Thus, endoglin + MPs from 8-week PAH rats stimulate an increase in whole cell ICAM-1 content in PAECs that is not associated with an increase in membrane content of ICAM-1.

## Discussion

Circulating MPs have recently garnered a great deal of interest as biomarkers. In PAH specifically, MPs are increased in total numbers. PECAM and VE-cadherin positive MPs correlate with increased mean pulmonary artery pressure and increased procoagulant MPs correlate with disease severity [2, 3]. Further, MPs induce expression of cellular adhesion molecules, such as ICAM-1, on systemic endothelium. While significant perivascular inflammation is observed in the remodeled and occluded pulmonary arteries of PAH, little is known about the mechanisms responsible for inflammatory cell recruitment [7, 8, 21–23]. Thus, we investigated whether circulating MPs from the Su/Hx/Nx rat model of severe PAH stimulate localized ICAM-1 on pulmonary endothelium. In the course of our studies we made three important observations. First, that circulating MPs from late-, but not early-stage PAH rats stimulated ICAM-1 expression on endothelium. Secondly, that this ICAM-1 expression was localized to the pulmonary arterial, and not microvascular, endothelium, and further that a select MP population, endoglin-positive MPs, were sufficient to induce intracellular ICAM-1 in pulmonary artery endothelium.

PAH is a progressively worsening disease that is likely dependent on multiple “hits” during manifestation. Our finding that 8-week, but not 3-week, MPs stimulated increased ICAM-1 may be indicative of the changing population of MPs present in the circulation as PAH progresses and pulmonary vascular lesions develop. Though we did not fully characterize the entire circulating MP populations, our finding that the endoglin-positive population makes up significantly more of the total MPs at 8 weeks as compared to 3 weeks supports this idea. These data also suggest that a longitudinal analysis of the various populations of MPs from endothelium may be important in identifying reporters of endothelial health in PAH.

Endoglin, as an accessory factor for TGF-β, reportedly plays a role in development of pulmonary arterial lesions [24, 25]. Endoglin-positive MPs may also be representative of a damaged or hyperproliferative endothelium such as that observed in PAH [3]. However, the impact of select populations of MPs on progression of the disease has not previously been investigated. We selected the endoglin + population and found that without the influence of the total MPs, endoglin + MPs alone were sufficient to induce increased ICAM-1 protein intracellularly. The effects of MPs, and even select populations of MPs from cell culture studies, have been examined on various endothelium, however this is the first report we are aware of that clearly reveals the impact of one subset of circulating MPs in PAH. Since PAH is a progressive and likely “multi-hit” disease, we can speculate that the endoglin + MPs or their contents induce ICAM-1 production, but a second “hit” is required for recruitment to the pulmonary artery endothelial membrane for functional recruitment and adhesion of inflammatory cells.

Endothelial heterogeneity within the pulmonary circulation is defined by location of the cells and their physiologic and pathologic responses [14, 26–28]. By testing endothelial cells derived from both the pulmonary arterial and microcirculations we could identify whether the circulating MPs affected a specific location. Importantly, in PAH the pulmonary vascular lesions, containing inflammatory cell infiltrates, develop in the pulmonary artery but not in the pulmonary microcirculation. The 8-week circulating MPs did not induce ICAM-1 expression in or on PMVECs, from the microcirculation. We cannot state explicitly that the MPs had *no* effect on the PMVECS, since we only examined ICAM-1 expression, and further studies would be necessary to determine whether there were influences on proliferation or other inflammatory molecules, however the implications are intriguing. The concept that MPs interact with cellular targets has been proposed, but rarely directly tested. These data leave open speculation on the mechanism of this “targeting”. Whether the MP possesses proteins or lipids necessary for interaction specifically with PAECs rather than PMVECs, or if the cell processes the interaction in a unique manner remains unknown. However, these are some of the first data to address MP effects using phenotypically distinct endothelium and to show differential responses. The implications of this work for PAH are that there are mechanisms inherent to circulating microparticles that influence the pulmonary artery, which do not stimulate the pulmonary microcirculation. A multitude of inflammatory factors have been reported to be increased in the circulation of PAH patients, but it has never been understood why the effects are only observed in specific vascular locations. MPs may contain the key to targeted vascular damage or even repair, however many studies will be necessary to answer these questions.

## Conclusions

In summary, our study highlights the impact of circulating PAH MPs selectively on the pulmonary artery endothelium leading to expression of both surface and intracellular ICAM-1. The increased intracellular ICAM-1 can be recapitulated with a selected population of PAH MPs, the endoglin + MPs, however this population does not increase surface expression. These are the first data to implicate PAH MPs, or a select population of circulating PAH MPs, in pulmonary vessel segment specific expression of ICAM-1. ICAM-1 likely plays an important role in recruitment of inflammatory cells to pulmonary vascular lesions in PAH.

## Additional file

Additional file 1: Figure S1. ICAM-1 expression is increased in pulmonary arteries of 8-week PAH rats. ICAM-1 is labeled green, and the blue are DAPI labeled nuclei. Intense staining is highlighted with red arrows. Control 8-week rats have limited ICAM-1 staining in pulmonary arteries and alveolar structures. Eight-week PAH rats have significant ICAM-1 staining in the airway, in crenelated or constricted pulmonary arteries, and lining open pulmonary arteries. Images are representative of *n* = 3 separate animals. (DOCX 5675 kb)

## Abbreviations

ICAM-1: Intercellular adhesion molecule-1
MPs: Microparticles
PAECs: Pulmonary artery endothelial cells
PAH: Pulmonary arterial hypertension
PMVECs: Pulmonary microvascular endothelial cells
